# Colorectal cancers utilize glutamine as an anaplerotic substrate of the TCA cycle in vivo

**DOI:** 10.1038/s41598-019-55718-2

**Published:** 2019-12-16

**Authors:** Yiqing Zhao, Xuan Zhao, Vanessa Chen, Ying Feng, Lan Wang, Colleen Croniger, Ronald A. Conlon, Sanford Markowitz, Eric Fearon, Michelle Puchowicz, Henri Brunengraber, Yujun Hao, Zhenghe Wang

**Affiliations:** 1 Department of Genetics and Genome Sciences, Case Western Reserve University, 10900 Euclid Avenue, Cleveland, Ohio 44106 USA; 2 Case Comprehensive Cancer Center, Case Western Reserve University, 10900 Euclid Avenue, Cleveland, Ohio 44106 USA; 3 Department of Nutrition, Case Western Reserve University, 10900 Euclid Avenue, Cleveland, Ohio 44106 USA; 4 Departments of Internal Medicine, Human Genetics, and Pathology, University of Michigan Medical School, Ann Arbor, MI 48109 USA; 5 Department of Medicine, Case Western Reserve University, 10900 Euclid Avenue, Cleveland, Ohio 44106 USA; 6 Seidman Cancer Center, University Hospitals Cleveland Medical Center, Cleveland, OH 44106 USA; 7 Present Address: Shanghai Cancer Institute, Shanghai Jiao-Tong University School of Medicine Renji Hospital, 25/Ln 2200 Xietu Road, Shanghai, 200032 P.R. China

**Keywords:** Cancer metabolism, Colorectal cancer

## Abstract

Cancer cells in culture rely on glutamine as an anaplerotic substrate to replenish tricarboxylic acid (TCA) cycle intermediates that have been consumed. but it is uncertain whether cancers *in vivo* depend on glutamine for anaplerosis. Here, following *in vivo* infusions of [^13^C_5_]-glutamine in mice bearing subcutaneous colon cancer xenografts, we showed substantial amounts of infused [^13^C_5_]-glutamine enters the TCA cycle in the tumors. Consistent with our prior observation that colorectal cancers (CRCs) with oncogenic mutations in the phosphatidylinositol-4,5-bisphosphate 3-kinase catalytic (PIK3CA) subunit are more dependent on glutamine than CRCs with wild type PIK3CA, labeling from glutamine to most TCA cycle intermediates was higher in PIK3CA-mutant subcutaneous xenograft tumors than in wild type PIK3CA tumors. Moreover, using orthotopic mouse colon tumors estalished from human CRC cells or patient-derived xenografts, we demonstrated substantial amounts of infused [^13^C_5_]-glutamine enters the TCA cycle in the tumors and tumors utilize anaplerotic glutamine to a greater extent than adjacent normal colon tissues. Similar results were seen in spontaneous colon tumors arising in genetically engineered mice. Our studies provide compelling evidence CRCs utilizes glutamine to replenish the TCA cycle *in vivo*, suggesting that targeting glutamine metabolism could be a therapeutic approach for CRCs, especially for PIK3CA-mutant CRCs.

## Introduction

It has been long recognized that cultured cancer cells can utilize glutamine as an anaplerotic substrate of the tricarboxylic acid (TCA) cycle^1^. Optimal operation of the TCA cycle allows the cancer cell to generate ATP and precursors for synthesis of lipids, nucleotides and other macromolecules^2–5^. Before entering the TCA cycle, glutamine is deamidated by glutaminases (GLSs), forming glutamate which is converted to α-ketoglutarate (α-KG), a TCA cycle intermediate^3,6^. A recent study found that cultured lung cancer cells use anaplerotic [^13^C_5_]-glutamine. However, when [^13^C_5_]-glutamine is infused *in vivo*, there was low labeling of the TCA cycle intermediates in the tumors^7^. These observations raise the possibility that glutamine dependency or addiction of cancers could be an artifact of cell culture, perhaps resulting from the high concentration of glutamine present in media.

We recently found that PIK3CA mutations render colorectal cancer (CRC) cells dependent on glutamine. PIK3CA, which encodes the catalytic subunit of phosphatidylinositol 3-kinase α (PI3Kα), is mutated in a wide variety of human cancers including ~30% of CRCs^8^. While PI3Ks convert phosphatidylinositol-4,5-bisphosphate (PIP2) to phosphatidylinositol-3,4,5-triphosphate (PIP3)^9,10^, the tumor suppressor protein PTEN catalyzes the reverse reaction^11^. The glutamine dependency of PIK3CA-mutant cancer cells is associated with upregulation of mitochondrial glutamate pyruvate transaminase 2 (GPT2)^12^, which converts glutamate to α-KG. Moreover, we demonstrated that aminooxyacetate (AOA), a pan-aminotransferase inhibitor, suppresses xenograft tumor growth of PIK3CA-mutant CRC, but not PIK3CA wild type (PIK3CA WT) CRC^12^. Interestingly, loss of PTEN also make breast cancers dependent on glutamine^13^. These observations indicate that the PI3K pathway plays a critical role in modulating glutamine metabolism in certain cancer types.

We previously showed that glutamine is anaplerotic in CRC cells in tissue culture^12^. Here, we infused [^13^C_5_]-glutamine in mice with subcutaenous or, orthotopic xenografts, and in mice genetically engineered to develop colon cancer. We found that infused [^13^C_5_]-glutamine labels the TCA intermediates of the tumor *in vivo*.

## Results

### [^13^C_5_]-glutamine in mouse plasma plateaus at 4 hours after infusion

**Figure 1:**
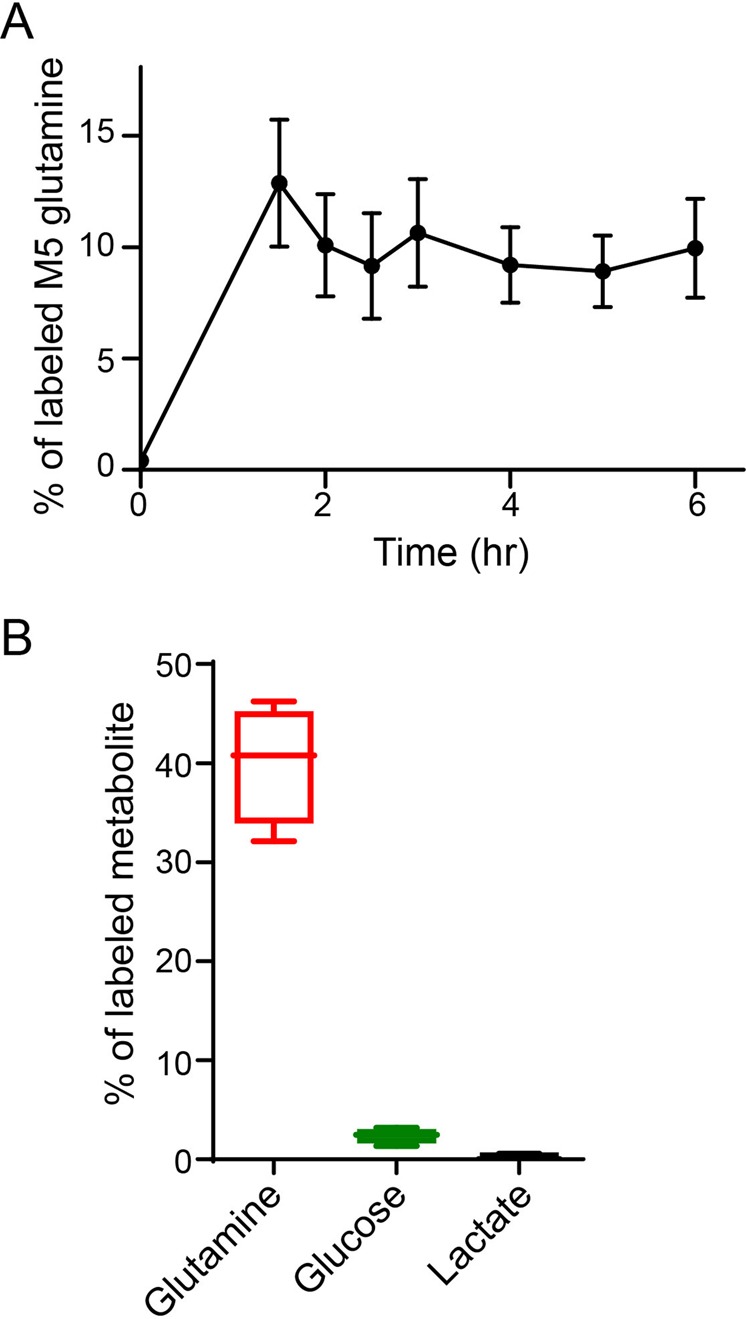
Kinetics of [^13^C_5_]-glutamine infusion in plasma. (**A**) Time course of labeled glutamine in mouse plasma. Mice (n = 4) were infused with [^13^C_5_]-glutamine as described in detail in the methods section. Plasma was taken at the indicated times and percentage of [^13^C_5_]-glutamine was measured by GC-MS. (**B**) Glutamine-derived lactate and glucose are negilible. Mice (n = 4) were infused in [^13^C_5_]-glutamine for 4 hours. Percentages of labled glutamine, lactate and glucose in plasma are shown.

To obtain an optimal time window for *in vivo* glutamine tracing, we infused mice with a bolus of 18.6 μmole/g of [^13^C_5_]-glutamine followed by an infusion rate of 20 μmole/(g x hour) for six hr. Plasma samples were taken every 30 min to measure M5 isotopic enrichment of glutamine. As shown in Fig. 1A, the labeling of plasma glutamine reached relative stable levels at 4 hr. There was no detectable isotopic enrichment of plasma lactate (Fig. 1B), whereas isotopic enrichment of plasma glucose is very low (Fig. 1B). Moreover, 3 to 6 hr of glutamine infusion have been used by others^7,14^. We thus chose to infuse [^13^C_5_]-glutamine into mice for 4 hr for in-depth studies.

### CRCs utilize glutamine as an anaplerotic substrate of the TCA cycle in subcutaneous xenograft tumor models

**Figure 2:**
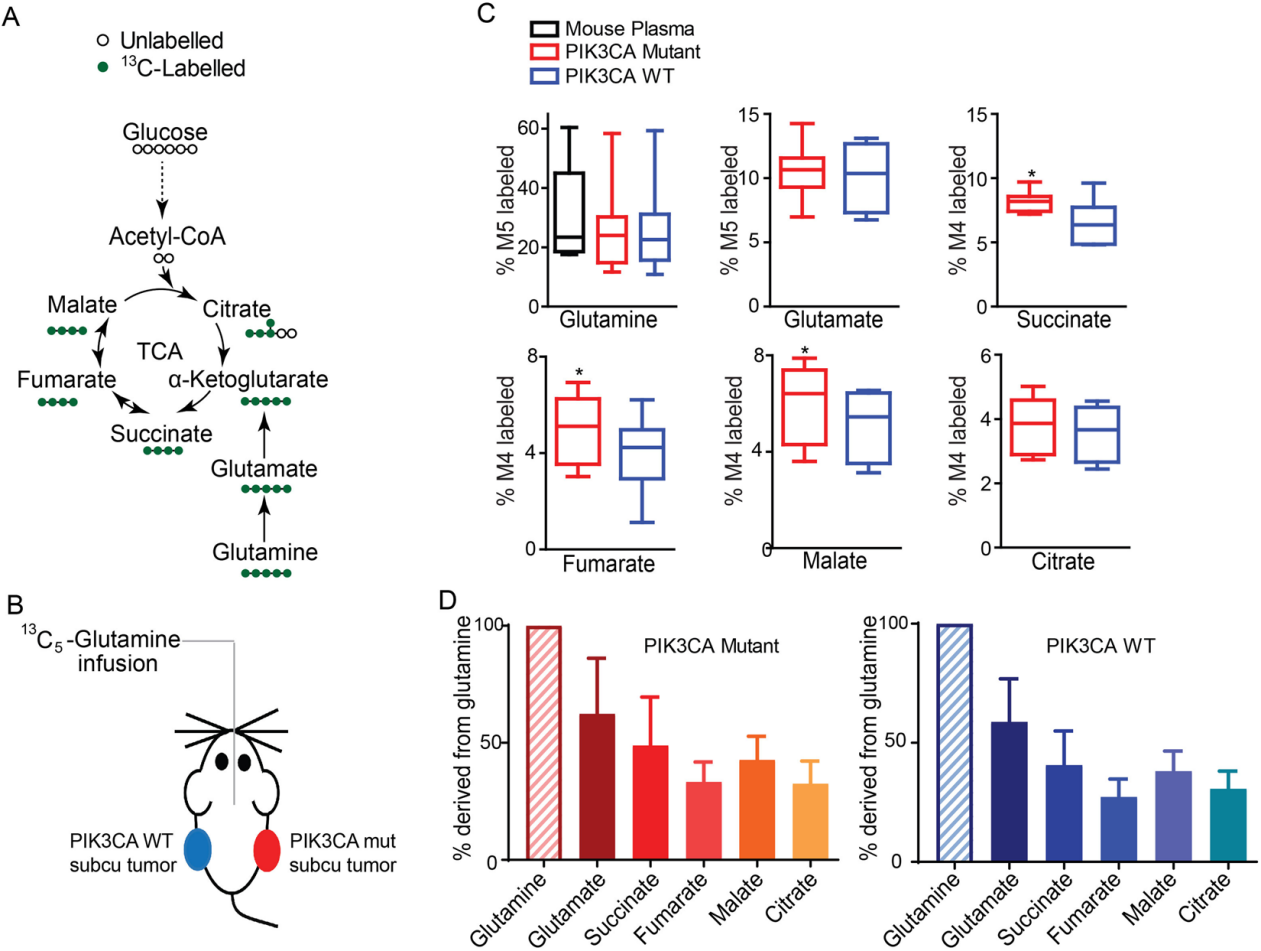
More [^13^C_5_]-glutamine enters the TCA cycle in PIK3CA mutant tumors in subcutanous xenograft models. (**A)** Schematic diagram of glutamine and its metabolites in the TCA cycle. **(B)** Schematic diagram of mice bearing subcutanous (subcu) xenograft tumors infused with [^13^C_5_]-glutamine. Isogenic HCT116 PIK3CA WT only cells, in which the mutant allele is knocked out, were injected into left flanks of nude mice, whereas HTCT116 PIK3CA mutant only cells, in which the WT allele was knocked out, were injected into the right. Two weeks post-injection, mice (n = 8) bearing similar size tumors in the two flanks were surgically catheterized for [^13^C_5_]-glutamine infusion. **(C)** More glutamine enters the TCA cycle in HTC116 PIK3CA mutant tumors than in the isogenic WT tumors. The indicated metabolite was measured by GC-MS and the percentage of the ^13^C-labeled metabolite in the total pool was calculated. *p < 0.05, the Student’s t test. **(D)** A significant fraction of glutamine enters the TCA cycle in xenograft tumors. Percentages of total ^13^C-labeled glutamate, succinate, fumarate, malate and citrate are normalized to total ^13^C-labeled glutamine and plotted.

We first traced glutamine metabolites in multiple nude mice carrying xenograft tumors formed by isogenic HCT116 PIK3CA-WT-only CRC cells (where the mutant PIK3CA allele was inactivated) in the left flank or PIK3CA-mutant-only (with the PIK3CA WT allele inactivated) CRC cells^15^ in the right flank (Fig. 2A,B). Consistent with our glutamine tracing data in tissue culture cells^12^, the labeling from glutamine of most of TCA cycle intermediates was higher in PIK3CA-mutant tumors than in PIK3CA-WT tumors (Fig. 2C). In contrast, the labeling from glucose of the TCA cycle intermediates was not different between PIK3CA-mutant tumors and PIK3CA-WT tumors (Fig. S1). We then compared the total labeling of each metabolite to the total labeling of glutamine. As shown in Fig. 2D, the labeling of glutamate and succinate were 60% and 40% of the labeling of glutamine, respectively. Also, the labeling of fumarate, malate and citrate were 30% to 40% of the labeling of glutamine (Fig. 2D). These data show that glutamine is a major anaplerotic substrate of the TCA in colorectal xenograft tumors.

### CRCs utilize glutamine as an anaplerotic substrate of the TCA cycle in orthotopic xenograft tumor models

**Figure 3:**
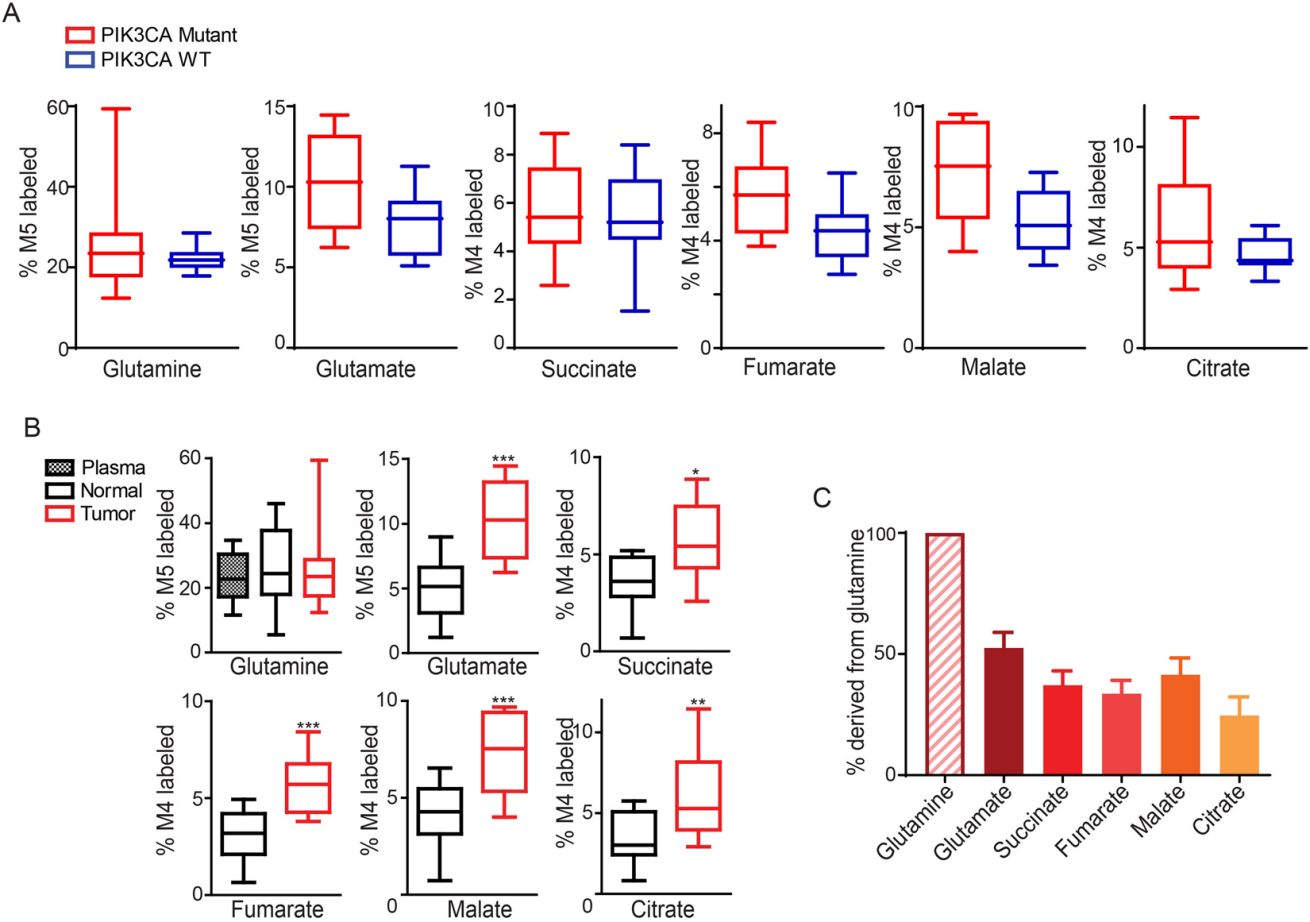
More [^13^C_5_]-glutamine enters the TCA cycle in PIK3CA mutant tumors than adjacent normal tissue in orthotopic xenograft models. [^13^C_5_]-glutamine tracing in orthotopic xenograft tumors established from WT-only (7 mice) and mutant-only (8 mice) cells. Two pieces (~1 mm^3^) of subcutaneous xenografts were sutured into the cecum serosa of nude mice. One day after the surgery, mice were infused with [^13^C_5_]-glutamine. The M5 enrichment of glutamine and the M4 enrichments of metabolites directly derived from M5 glutamine in WT- and mutant-only tumors are shown in (**A**). The M5 enrichment of glutamine and the M4 enrichments of metabolites directly derived from M5 glutamine in HCT116 mutant-only tumors and adjacent cecum tissues are shown in (**B**). Percentages of total ^13^C-labeled metabolited normalized to total ^13^C-labeled glutamine in the mutant-only tumors are plotted in (**C**). *p < 0.05, **p < 0.01, ***p < 0.001; the Student’s t test.

Next, we traced the labeling of glutamine in metabolites in orthotopic colon cancer models, which provide an organ-relevant tumor microenvironment. Although not statistically significant, the labeling of glutamine-derived metabolites was higher in tumors established from HCT116 PIK3CA-mutant only cells than in tumors established from the HCT116 PIK3CA-WT only cells (Fig. 3A). Nonetheless in the tumors, the labeling of metabolites from glutamine was greater than in the adjacent normal cecum tissues (Fig. 3B). It seems that glutamine is metabolized in a similar fashion at different locations in colon, as similar amounts of isotopic enrichments of glutamine, glutamate and the TCA intermediates were observed in cecum and colon tissues (Fig. S2). As with the subcutaneous xenograft models, glutamine substantially labeled glutamate and TCA cycle intermediates in orthotopic xenograft tumors (Fig. 3C).

### A patient-derived CRC xenograft utilizes glutamine as an anaplerotic substrate of the TCA cycle in an orthotopic model

**Figure 4:**
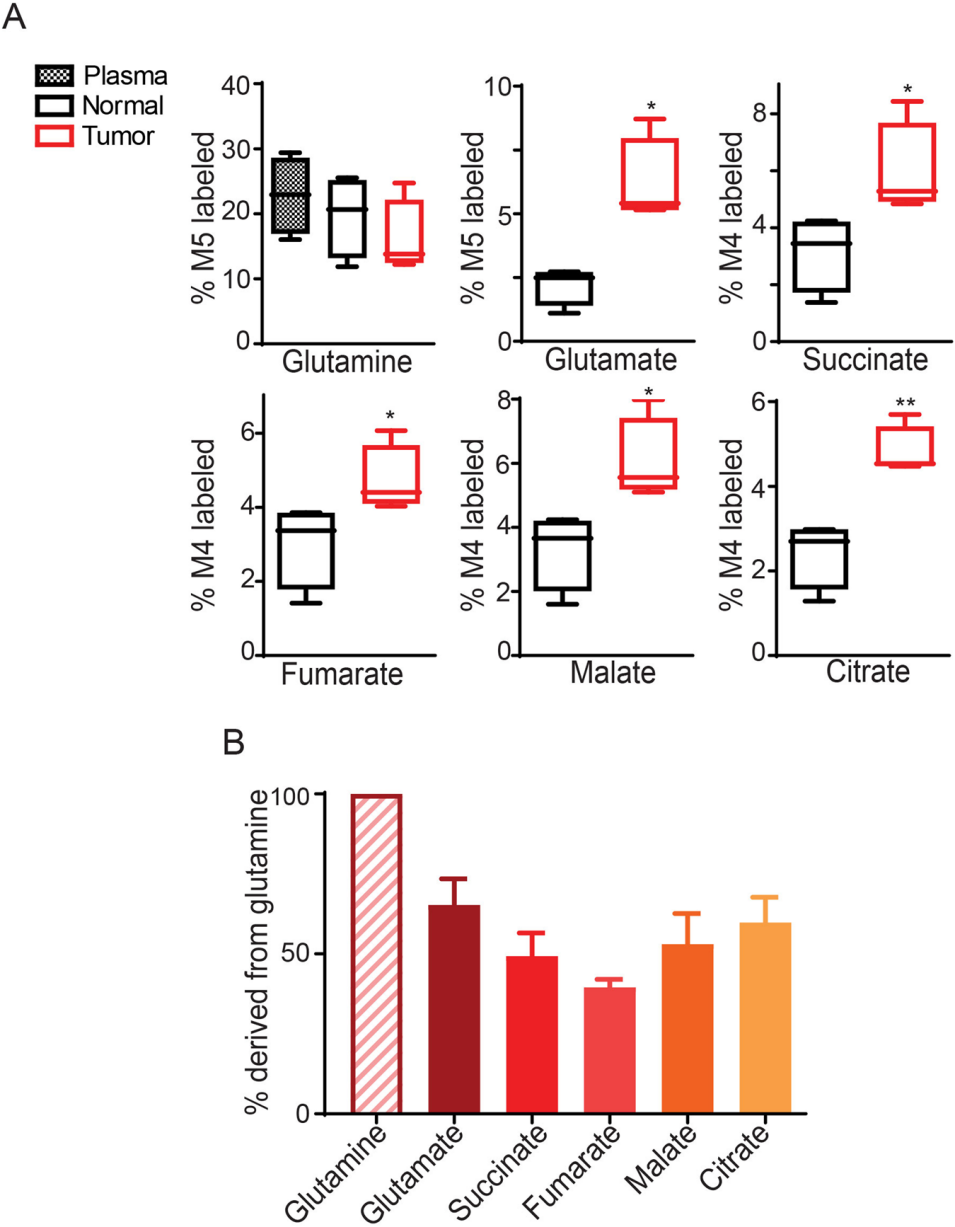
More [^13^C_5_]-glutamine enters the TCA cycle in PIK3CA mutant tumors than adjacent normal tissue in orthotopic patient-derived xenografts. (**A**,**B**) [^13^C_5_]-glutamine tracing in orthotopic xenograft tumors established from a colon cancer patient-derived xenograft. The M5 enrichment of glutamine and the M4 enrichments of metabolites directly derived from M5 glutamine are shown in (**A**). Percentages of total ^13^C-labeled metabolited normalized to total ^13^C-labeled glutamine in tumors are plotted in (**B**). *p < 0.05, **p < 0.01; the Student’s t test.

It is generally believed that patient-derived xenografts (PDXs) recapitulate the heterogeneity of human cancer better than xenografts established from cancer-derived cell lines^16^. We thus performed [^13^C_5_]-glutamine tracing in mice bearing orthotopic tumors established from a PDX. As shown in Fig. 4A, the enrichments of M5-labled glutamate and M4-labeled succinate, fumarate, malate and citrate were significantly higher in the PDX tumors than in the adjacent cecum tissues. Moreover, the TCA cycle intermediates were labeled at 30 to 50% of the level of glutamine (Fig. 4B).

### Spontaneously arising mouse colon tumors carrying PIK3CA alterations utilize glutamine as an anaplerotic substrate of the TCA cycle

**Figure 5:**
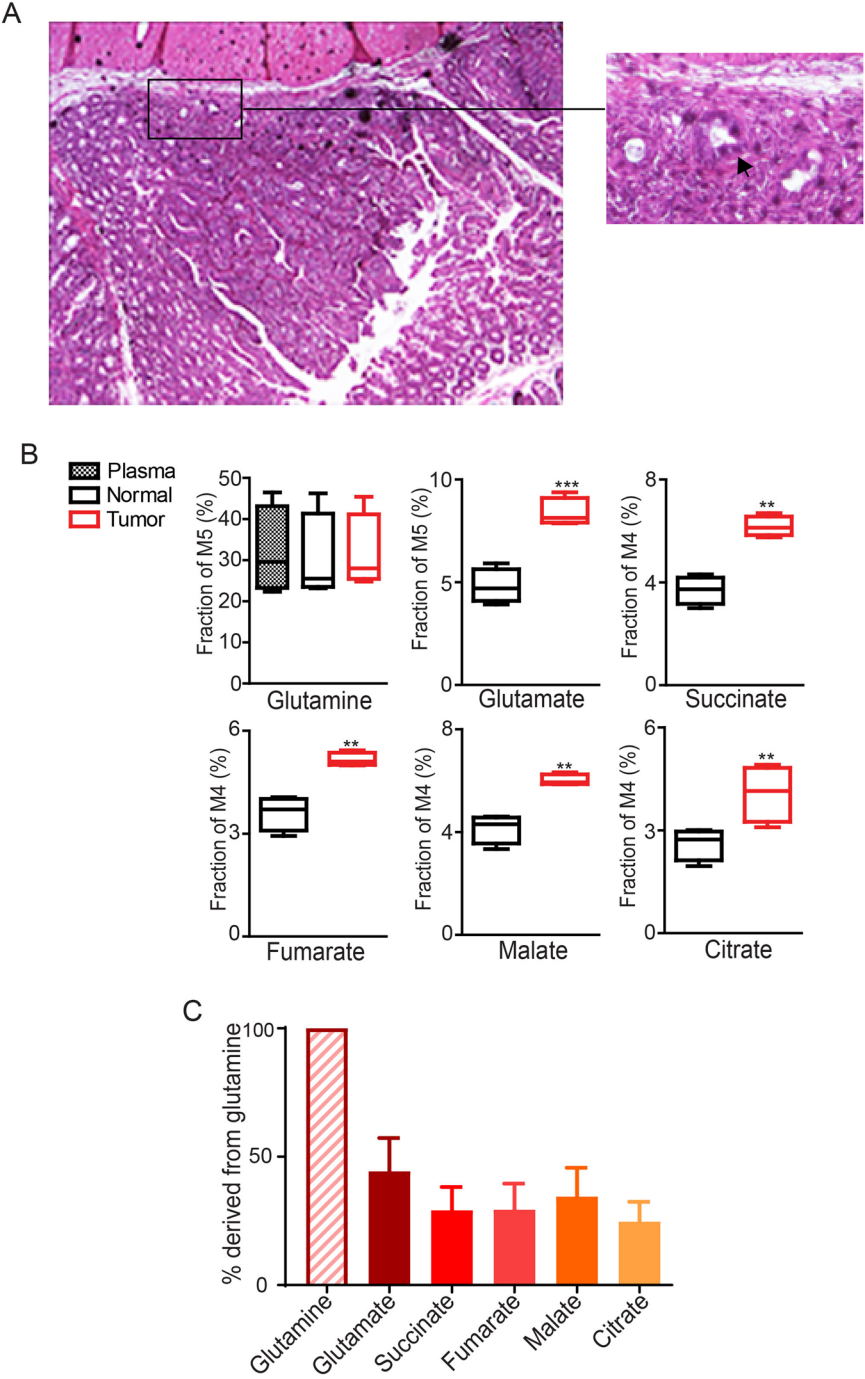
More [^13^C_5_]-glutamine enters the TCA cycle in PIK3CA mutant tumors than adjacent normal tissue in genetically engineered mice. *CDX2P-CreER*^*T2*^ *Apc*^*flox*/+^ *Kras*^*LSL-G12D*/+^ *Pik3ca*^*LSL-E545K*/+^ mice were treated with tamoxifen to conditionally express the Kras and Pik3ca oncogenes and to delete Apc. Two moths post-treatment, mice were infused with [^13^C_5_]-glutamine. Representative image of H & E staining of a colon tumor is shown in (**A**). Arrow indicates a tumor invaded to the muscle layer. The M5 enrichment of glutamine and the M4 enrichments of metabolites directly derived from M5 glutamine are shown in (**B**). Percentages of total ^13^C-labeled metabolited normalized to total ^13^C-labeled glutamine in tumors are plotted in (**C**). *p < 0.05, **p < 0.01, ***p < 0.001; the Student’s t test.

To determine how spontaneous colon tumors metabolize glutamine in immune-competent mice, we infused [^13^C_5_]-glutamine in *CDX2P-CreER*^*T2*^ *Apc*^*flox*/+^ *Kras*^*LSL-G12D*/+^ *Pik3ca*^*LSL-E545K*/+^ mice, which developed multiple advanced non-invasive and invasive colon tumors in the cecum and proximal colon within two months after tamoxifen administration (Fig. 5A). Although the labeling of glutamine was similar in tumor tissues and adjacent normal colon tissues, the enrichments of M5-labled glutamate and M4-labeled succinate, fumarate, malate and citrate were significantly higher in tumors than normal tissues (Fig. 5B). Furthermore, TCA cycle intermediates were labeled at 30% of the level of glutamine (Fig. 5C). Together, these data demonstrate that anaplerosis from glutamine is more intense in colon tumors than in normal colon tissues *in vivo* and that a substantial fraction of TCA cycle intermediates are derived from glutamine.

## Discussion

Our *in vivo* glutamine infusion data in subcutaneous, orthotopic and genetically engineered colon tumor models clearly demonstrate that (1) a substantial fraction of glutamine in tumors enters the TCA cycle and (2) relative anaplerosis from glutamine is more intense in the tumors than in adjacent normal colon tissues. While our data demonstrate that glutamine is a major anaplerotic substrate for CRCs, Vander Heiden and colleagues have previously reported that only minimal amounts of glutamine enter the TCA cycle in spontaneous lung tumors arising from activation of a mutant Kras allele in mouse lung epithelium^7^. In orthotopic glioblastoma models, Marin-Valencia and co-workers reported that the three glioblastoma PDXs they examined utilized glucose, not glutamine, to fuel the TCA cycle^14^. In contrast to the findings in these two studies, using hyperpolarized [1-^13^C] glutamine magnetic resonance imaging, Salamanca-Cardona and colleagues reported that glutamine, but not glucose, enters the TCA cycle to generate 2-hydroxyglutarate *in vivo* in patient-derived chondrosarcoma with IDH1 or IDH2 mutation^17^. Of interest, a recent study indicated that glutamine is the predominant carbon source for the TCA cycle for pancreas, intestine and spleen, whereas glucose or lactate is the major carbon source for the TCA cycle in brain, lung, and other tissues^18^. The seemingly discordant results in the literature for glutamine utilization by cancers may reflect differences among in tissue/cell context, genetic and epigenetic differences in different tumor types, and potentially various other factors and mechanisms. Consistently, we did not detect any [^13^C_6_]-glucose in the CRC xenograft tumors, precluding us from calculating the percentages of TCA cycle metabolites derived from glucose.

Our [^13^C_5_]-glutamine tracing data in tissue culture and subcutanous xenograft tumors show that isogenic PIK3CA-mutant CRCs utilize more anaplerotic glutamine than PIK3CA-WT counterparts. In the orthotopic models, the enrichments of TCA cycle intermediates are higher in PIK3CA-mutant than in the WT tumors, but they are not statistically significant. This discrepancy may due to the small sample size and experimental variabilities of the orthotopic models. In the subcutanous xenograft models, isogenic PIK3CA WT and mutant cells are implanted pair-wise into the same mouse, whereas in the othotopic models each mouse bears either a PIK3CA WT or a mutant tumor. Thus, the experimental conditions in the subcutanous xenograft models are better controlled than in the orthotopic models. We believe that the data obtained in the subcutanous models reflect the intrinsic difference of glutamine metabolism between PIK3CA WT and mutant tumors, that is, PIK3CA mutant tumors are more dependent on anaplerotic glutamine. However, we could not completely rule out the possibility that the difference in glutamine anaplerosis between PIK3CA mutant and WT tumors is a consequence of different tumor growth rate. Nonetheless, we found that, as with AOA, CB-839 preferentially inhibits xenograft tumor growth of PIK3CA mutant, but not WT, CRC xenograft tumor growth^19^. Moreover, the combination of CB-839 and 5-FU induced tumor regression in three different PIK3CA mutant CRC xenograft models^19^. These exciting results prompted to conduct a phase I/II clinical trial of combinational of CB-839 with capecitabine, an oral prodrug of 5-FU, (https://clinicaltrials.gov/ct2/show/NCT02861300). The phase I trial demonstrated that the drug combination is well tolerated at biologically-active doses^20^. Consistent with the preclinical data, an exploratory analysis of time on treatment and progression free survival suggests that PIK3CA-mutant CRC patients may derive greater benefit from this treatment strategy as compared to PIK3CA WT CRC patients^20^.

Although our studies focus on CRCs, *in vivo* glutamine dependency may be a general phenomenon for various tumor types, as recent studies demonstrate that a variety of tumor types including breast, pancreatic, kidney cancers as well as acute myeloid leukemia are sensitive to a glutaminase inhibitor, CB-839, *in vivo*^21–24^. Clinical trials of combination of CB-839 with various therapeutic agents are ongoing in patients with renal cell carcinomas (RCC), melanoma, or non-small cell lung cancer (https://www.cancer.gov/about-cancer/treatment/clinical-trials/intervention/glutaminase-inhibitor-cb-839). Interestingly, Combinations of CB-839 with cabozantinib, everolimus have shown promising results in a phase I clinical trial in RCC patients^25,26^. Further studies should help to better define factors and mechanisms accounting for glutamine dependency *in vivo* in different cancer types and the ramifications arising from such a dependency for improving treatment outcomes in cancer patients.

## Materials and Methods

### Cell culture

HCT116 CRC cell lines were obtained from ATCC (catalog # CCL-247). The isogenic HCT116 PIK3CA WT and mutant only cell lines were kind gifts from Dr. Bert Vogelstein at John Hopkins University. These cell lines were cultured in McCoy’s 5A medium containing 10% fetal bovine serum as described previously^27^. The tissue cultures were routinely checked to ensure free of mycoplasma contamination. The cell lines were authenticated by the Genetica DNA Laboratories using STR profiling.

### Animal models

Animal experiments were approved by the Case Western Reserve University Animal Care and Use Committee. All experiments were performed in accordance with relevant guidelines and regulations.

#### Subcutaneous xenograft

As described in^28^, 3 million cells were injected subcutaneously into the flanks of 6 to 8-week-old female athymic nude mice.

#### Orthotopic xenograft

Eight-week-old female nude mice were anesthetized by intraperitoneal injection of ketamine (104 mg/kg) and xylazine (9 mg/kg) and disinfected with iodine/alcohol prep pads. A laparotomy incision of approximately 1.0 cm was made in the skin just to the right of the abdomen midline. The cecum was pulled out of the abdomen and a piece of subcutaneous xenograft tumor (~1 mm^3^) was attached to the serosa of cecum using 6–0 silk sutures. The cecum was then put back in the abdominal cavity. The muscle layer and abdominal wall were closed with 4–0 Vicryl plus antibacterial violet sutures.

#### Genetically engineered mice

*Apc*^*flox/flox*^ mice^29^, *Pik3ca*^*LSL-E545K*/+^ mice^30^, and *Kras*^*LSL-G12D*/+^ mice^31^ have been previously described. *CDX2P-CreER*^*T2*^ transgenic mice^32^ were first intercrossed with *Apc*^*flox/flox*^ mice and *Pik3ca*^*LSL-E545K*/+^ mice to generate the *CDX2P-CreER*^*T2*^ *Apc*^*flox/flox*^ *Pik3ca*^*LSL-E545K*/+^ mice. These mice were then bred to *Kras*^*LSL-G12D*/+^ mice to produce the *CDX2P-CreER*^*T2*^ *Apc*^*flox*/+^ *Kras*^*LSL-G12D*/+^ *Pik3ca*^*LSL-E545K*/+^ mice. For Cre-mediated deletion of the *Apc* floxed allele and the lox-STOP-lox cassette (LSL) in *Kras*^*LSL-G12D*^ and *Pik3ca*^*LSL-E545K*^ mutant alleles, the mice were injected intraperitoneally with tamoxifen (100 mg/kg body weight; Sigma-Aldrich, St Louis, MO) once daily for 2 days. Two months post tamoxifen administration, the mice were infused with [^13^C_5_]glutamine to check the glutamine usage.

### Mouse infusion

Surgical procedures were similar to those established by the Mouse Metabolic Phenotyping Consortium^33^. Briefly, mice were anesthetized and a 2 cm skin incision was made on the right side of the neck. Blunt forceps were used to isolate a 5 mm section of the jugular vein and 4–0 silk suture was tied on both proximal and distal ends of the vessel. RenaSil Silicone Rubber Tubing (0.025″ OD × 0.012″ID) was inserted into the vein. The ends of the free catheter were tunneled under the skin to the back of the neck, and sealed with steel plugs.

One day after surgery, fasted mice were first infused by bolus (150 mM Glucose, 124 mM Glutamine in 150 mM NaCl solution) as 0.3 ml/20 g mice, and then followed by infusion solution (266 mM Glucose, 137 mM Glutamine in 150 mM NaCl solution) at an infusion rate of 0.3 ml/hour/20 g. Mice were sacrificed after 4 hours of infusion, and tissue and plasma were collected for metabolites analysis by GC-MS. For glutamine tracing, L-Glutamine-^13^C_5_ was applied instead of L-Glutamine. For glucose tracing, D-Glucose-^13^C_6_ was applied instead of D-Glucose.

### Metabolite assays

Frozen tissue was homogenized with metabolite extraction buffer (90% methanol and 10% PBS, pre-chilled in −80 °C). For 100 mg tissue, 1 ml of buffer was applied. 5 µM of heptadecanoic acid, 2.5 µM of [3,3,4,5,5,5-2H6]4-hydroxypentanoate and 2.5 µM of [2,2,3,3,4,4,5,5,6,6,7,7,7-2H13]heptanoate were added into the extraction buffer as internal standards. After centrifuging at 14,000 rpm for 15 min at 4 °C, the supernatant was collected and dried with nitrogen gas. TBDMS (MTBSTFA + TBDMCS, REGIS Technologies):Acetonitrile (2:1) were used for derivatization of metabolites at 65 °C for 1 hour. 1 µl of samples was injected into GC-MS (Agilent Technologies) for analysis. For the analysis of the fraction of C13 labeled metabolites, the total pool of each metabolite was set to 100%, C13 labeled metabolites isotopomer distribution (enrichment) indicated percentage of each isotopomer to total pool. To calculate percentage of C13 in total C pool for each metabolite, the formula of (1*M_1_% + 2*M_2_% + 3*M_3_% + … + n*M_n_%)/n was applied.

For glucose analysis with GC-MS, samples were processed with extraction buffer (90% methanol and 10% PBS, pre-chilled in −80 °C). The supernatant was collected and dried with nitrogen gas. To the dried residue, hydroxylamine hydrochloride (2.1 mg) in 100 µl of pyridine was added and the mixture heated at 90 °C for 30 min, then 75 µl of acetic anhydride was added and heated for an additional hour. The reaction mixture was cooled, partitioned between water and methylene chloride layer. The methylene chloride layer was then transferred to a vial, dried in a stream of air and reconstituted with 50 µl of ethyl acetate.

### H&E staining

H & E staining was performed as described^34^. Briefly, formalin-fixed, paraffin-embedded tissue sections (5 μm) were pre-warmed in a 60 °C incubator for 1 h 15 min, then were deparaffinized in xylene and rehydrated in ethanol gradient. Sections were stained with hematoxylin and eosin (H&E).

### Statistical analysis

GraphPad Prism software was used to create the graphs. Data are plotted as mean ± SEM. We applied the *t* test to compare the means between two groups, assuming unequal variances.

## Supplementary information

Supplementary Figures

## Data Availability

All data generated or analysed during this study are included in this published article (and its Supplementary Information Files).
