# Supplementary material for: Colorectal cancers utilize glutamine as an anaplerotic substrate of the TCA cycle in vivo
Source: Sci Rep. 2019 Dec 16;9:19180. doi: 10.1038/s41598-019-55718-2 (PMC6915720; doi:10.1038/s41598-019-55718-2)
Supplement: Supplementary file 1 — Supplementary Figures [file 41598_2019_55718_MOESM1_ESM.pdf]

Colorectal cancers utilize glutamine as an anaplerotic substrate of the TCA cycle in vivo

Yiqing Zhao[1,2], Xuan Zhao[1,2], Vanessa Chen[1,3] Ying Feng[4], Lan Wang[3], Colleen C. Croniger[3], Ronald A. Conlon[1,2], Sanford Markowitz[1,2,5,6], Eric Fearon[4], Michelle A. Puchowicz[3], Henri Brunengraber[3], Yujun Hao[1,2, 7]*, and Zhenghe Wang[1,2]*

[1]Department of Genetics and Genome Sciences,

[2]Case Comprehensive Cancer Center,

[3]Department of Nutrition,

[5]Department of Medicine,

Case Western Reserve University,

10900 Euclid Avenue, Cleveland, Ohio 44106.

[4]Departments of Internal Medicine, Human Genetics, and Pathology, University of Michigan Medical School, Ann Arbor, MI 48109

[6]Seidman Cancer Center, University Hospitals Cleveland Medical Center, Cleveland, OH 44106.

[7] Current address: Shanghai Cancer Institute, Shanghai Jiao-Tong University School of Medicine Renji Hospital,

25/Ln 2200 Xietu Road, Shanghai 200032, P. R. China.

* To whom correspondence should be addressed. Emails: zxw22@case.edu (lead contact) and yjhao@shsci.org

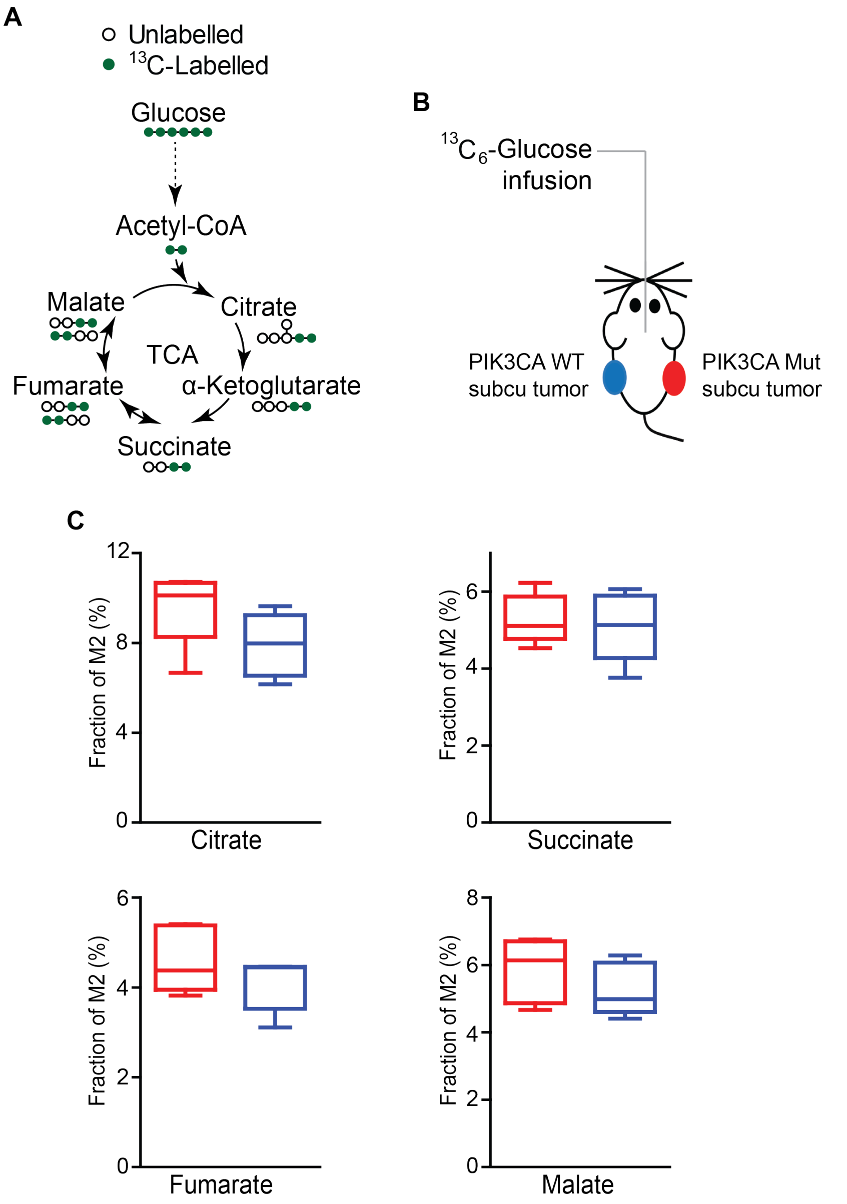

Supplementary Figure 1 (A) Schematic diagram of glucose and its metablites in the TCA cycle. **(B)** Schematic diagram of mice bearing subcutanous (subcu) xenograft tumors infused with [$^{13}C_6$]-glucose. Isogenic HCT116 PIK3CA WT only cells, in which the mutant allele is knocked out, were injected into left flanks of nude mice, whereas HTCT116 PIK3CA mutant only cells, in which the WT allele was knocked out, were injected into the right. Two weeks post-injection, mice (n = 8) bearing similar size tumors in the two flanks were surgically catheterized for [$^{13}C_6$]-glucose infusion. (**C**) The indicated metabolite was measured by GC-MS and the percentage of the ^{13}C-labeled metabolite in the total pool was calculated.

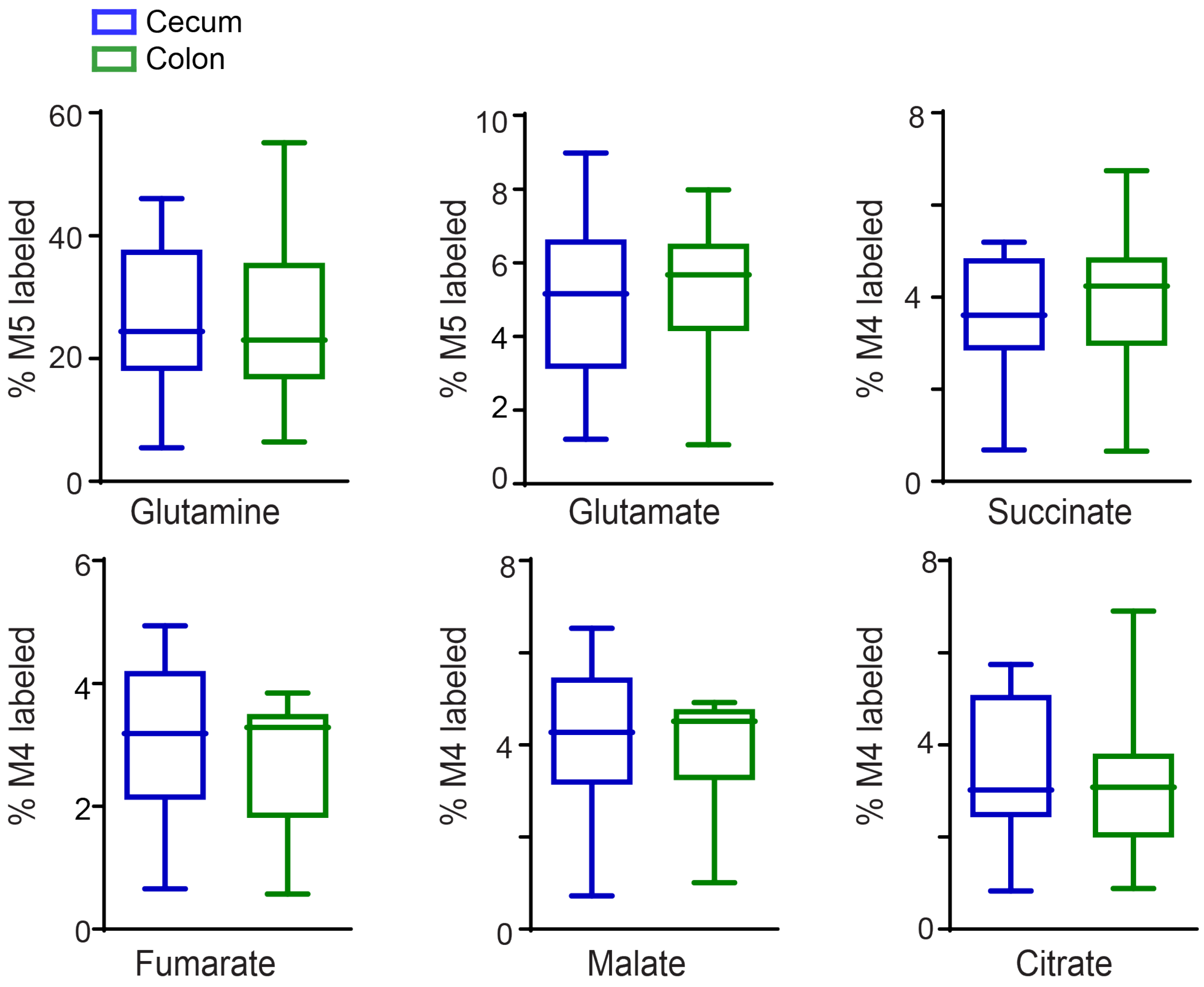

Supplementary Figure 2. The M5 enrichment of glutamine and the M4 enrichments of metabolites directly derived from M5 glutamine in cecum and colon (8 mice) are shown.
